# Genetic alterations of chromosomes, p53 and p16 genes in low- and high-grade bladder cancer

**DOI:** 10.3892/ol.2014.2108

**Published:** 2014-05-02

**Authors:** DENIZ ABAT, OSMAN DEMIRHAN, NIHAL INANDIKLIOGLU, ERDAL TUNC, SEYDA ERDOGAN, DENIZ TASTEMIR, INAYET NUR USLU, ZUHTU TANSUG

**Affiliations:** 1 Department of Urology, Faculty of Medicine, Çukurova University, 01330 Adana, Turkey; 2 Department of Medical Biology, Faculty of Medicine, Çukurova University, 01330 Adana, Turkey; 3 Department of Pathology, Faculty of Medicine, Çukurova University, 01330 Adana, Turkey; 4 Vocational School of Health Services, Adıyaman University, 02040 Adıyaman, Turkey

**Keywords:** low grade, high grade, bladder carcinoma, chromosomal aberrations, p16, p53, fluorescence *in situ* hybridization

## Abstract

A majority of patients with bladder cancer present with superficial disease and subsequently, some patients show progression to muscle invasive or metastatic disease. Bladder cancer has a complex genetic process and identification of the genetic alterations which occur during progression may lead to the understanding of the nature of the disease and provide the possibility of early treatment. The aim of the present study was to compare the structural and numerical chromosomal differences and changes in the p16 and p53 genes between low-grade (LG) and high-grade (HG) bladder cancer (BC) using cytogenetic and molecular cytogenetic methods. Between March 2009 and March 2010, cytogenetic analyses were carried out on tumor and blood samples in 34 patients with transitional cell type BC, and on blood samples of 34 healthy patients as a control group. Fluorescence *in situ* hybridization probes for the p16 and p53 genes were also used to screen the alterations in these genes in 32 patients with BC. The patients were divided into two groups (LG and HG) and the findings were compared. A total of 11 (32.3%) patients exhibited LGBC, 22 (64.7%) exhibited HGBC and one (3%) patient exhibited carcinoma *in situ*. There were no differences between the LGBC and HGBC groups according to the number of chromosomal aberrations (P=0.714); however, differences between alterations of the p16 and p53 genes were significant (P=0.002 and P=0.039). Almost all structural abnormalities were found to be located to the 1q21, 1q32, 3p21 and 5q31 regions in patients with HG tumors. In conclusion, the p16 and p53 genes were altered more prominently in patients with HG tumors compared with LG tumors. The structural abnormalities in the 1q21, 1q32, 3p21 and 5q31 regions were observed more frequently in patients with HG tumors. These regions may play significant roles in the progression of BC, but further studies are required to find candidate genes for a panel of BC.

## Introduction

An estimated 386,300 new cases and 150,200 fatalities from bladder cancer (BC) occurred worldwide in 2008 (1). BC has a number of known risk factors, including age, cigarette smoking, exposure to chemicals, chronic infections or irritations and exposure to pelvic radiation. However, numerous patients with BC have no history of exposure to carcinogens (2). The identification of genetic events during tumorigenesis may lead to an understanding of the genetic mechanism underlying BC.

In total, ~75% of patients present with superficial disease (Ta and T1) and 20% with T2 or higher disease. Overall, 70% of treated tumors recur, with 30% of recurrent tumors progressing to metastatic disease of the non-muscle-invasive lesions. Approximately 10% of low-grade (LG) papillary tumors subsequently develop muscle-invasive or metastatic cancer, whereas roughly a third of high-grade (HG) tumors progress, if not already, to muscle-invasive at the time of diagnosis (3). Therefore, the determination of the ideal biomarkers for predicting progression to invasion or metastatic disease is important.

The molecular and genetic changes in urothelial carcinoma (UC) of the bladder are grouped into three processes: i) Chromosomal alteration, which activates the initial carcinogenic event; ii) tumor proliferation, due to a loss of cell-cycle regulation and derangements in normal apoptotic turnover; and iii) metastasis, which involves the initial tumor migration and other processes, including angiogenesis and loss of cell adhesions (4). Since studies have revealed the association between genetic changes and BC, numerous genes have been studied for their connection to BC (5–7). It is known that p53 plays a key role in the regulation of the cell cycle, and mutations in p53 result in chromosomal instability. Alterations in the p53 gene are more frequently observed in invasive HG tumors compared with LG tumors (6). The cyclin-dependent kinase inhibitors p21 and p16 are correlated with an increased disease recurrence and progression. Additionally, the genesis and/or progression of BC has been shown to be a consequence of genetic instability, and chromosomes 3, 7, 9 and 17 are frequently involved in uroepithelial oncogenesis (8,9).

In the present study, cytogenetic methods and fluorescence *in situ* hybridization (FISH) were used to investigate the frequencies of chromosomal aberrations (CAs) and alterations (amplifications and deletions) of the p53 and p16 genes, alone or in combination, in Turkish patients with BC. The results were compared between cases of HGBC and LGBC.

## Materials and methods

### Patients

Between March 2009 and March 2010, following approval of the study by the ethics committee of the Medical Faculty of Çukurova University (Adana, Turkey), blood and tissue samples were collected from 34 patients with BC. Written informed consent was obtained from all patients. Tissue samples were removed by transurethral resection or from radical cystectomy specimens, and blood samples were drawn simultaneously during these surgical procedures. A small piece of the tumor sample was obtained for genetic study. The remainders of the tissue samples were evaluated in the Department of Pathology, Çukurova University (Adana, Turkey) by the same pathologist. Structural and numerical abnormalities of chromosomes were detected in the blood and tissue samples from patients with BC by cytogenetic methods. The blood samples from 34 healthy patients were collected and analyzed as the control group. The p16 and p53 genes were also identified in the bladder tumor samples using FISH. The numbers of CAs, including deletion, amplification, fragility, chromosome break, chromatin break and translocation, were compared among the patient and control groups. The patients with BC were divided into two groups: LG and HG. This was performed according to the histopathological type of tumors present, based on World Health Organisation histological criteria (10). Subsequently, the two groups were compared according to age, body mass index (BMI), smoking history, number of chromosomal abnormalities and differences in p16 and p53 genes. Finally, the values were assessed using statistical methods.

### Cytogenetic examination

The peripheral blood from 34 patients was obtained for culture and FISH studies. The expression of folate-sensitive fragile sites (FSs) and cytogenetic abnormalities (CAs) in each sample was examined in the genetic laboratory of the Department of Medical Biology and Genetics, Faculty of Medicine, Çukurova University. A 0.3-ml blood sample was incubated at 37°C for 72 h in two types of media; RPMI-1640 (Sigma-Aldrich, St. Louis, MO, USA) and M199 without folic acid (Biological Industries Israel Beit-Haemek, Ltd., Kibbutz Beit-Haemek, Israel). Standard cytogenetic techniques were used for harvesting and slide preparation. The slides were first stained only with Giemsa prior to the examination to avoid missing any gaps. For a detailed analysis of the FSs, a few slides were prepared by GTG-banding, and 50 metaphases were scored for each assay. A CA was defined when it was present in 1% of the cells analyzed and in ≥50% of the individuals studied (11). All gaps and breaks were recorded and localized according to the International System for Human Cytogenetic Nomenclature (1995) (12). The classification of CAs was carried out according to the nomenclature established in the 11th International Workshop on Human Gene Mapping (13).

### Tumoral tissues

Bladder tumor samples were obtained from 32 patients by transurethral resection or from radical cystectomy specimens. All samples were mechanically minced and enzymatically disaggregated by digestion with trypsin-EDTA (Biological Industries Israel Beit-Haemek Ltd.) for 1 h. Following the digestion, BioAMF1 medium (Biological Industries Israel Beit-Haemek Ltd.) supplemented with supplement, penicillin-streptomycin and gentamycin (all Biological Industries Israel Beit-Haemek Ltd.) was used for culture. A long-term cell culturing method was performed for proliferation of tumor and normal cells. Once enough proliferation (average, 10 days) had occurred, standard cytogenetic techniques were used for harvesting and slide preparation. GTG-banding was achieved by trypsin-Giemsa treatment. The karyotype was determined by analyzing ≥25 metaphases from the normal and tumor bladder epithelium cells for each individual patient. If there were not enough metaphases observed, the slides were evaluated. For eliminating inherited CAs, lymphocyte cultures were also performed and 25 metaphases were counted for each patient.

### Slide preparation and FISH analysis

Cytogenetic analysis of BC cells has remained difficult as these cells have a risk of infection and limited proliferative capacity *in vitro*, which precludes analysis by metaphase cytogenetics. Therefore, interphase FISH was used to study p53 and p16 genes in non-dividing cells. Standard cytogenetic techniques were used for harvesting and preparation of slides for FISH (14). To observe the p53 and p16 genes, bladder tissues from 32 patients were examined by interphase FISH. Poseidon Repeat-Free FISH Probe p16 (on chromosome 9p21/9q21) and Poseidon Repeat-Free FISH Probe p53 (on chromosome 17p13/SE 17) probes purchased from Kreatech Diagnostics (Amsterdam, The Netherlands) were used.

### Statistical analysis

Comparisons between groups were applied using Student’s t-test and one-way analysis of variance for normally distributed data. The Mann-Whitney U-test and Kruskal-Wallis test were used to compare data that were not normally distributed. The categorical variables between groups were analyzed using the χ^2^ test. Results are presented as the mean ± standard deviation and the median (range). P<0.05 was considered to indicate a statistically significant difference. Statistical analyses were performed using SPSS, version 18.0 (SPSS, Inc., Chicago, IL, USA).

## Results

### Demographic data of the patients

A total of 30 (88.2%) male and four (11.2%) female patients with BC were recruited for the present study, with a mean age of 60.6±14.2 years (range, 26–81 years). Histopathological examinations revealed that 11 (32.3%) patients had LGUC, 22 (64.7%) patients had HGUC and one (3%) patient had carcinoma *in situ* (CIS). The patient with CIS was added to the HG-tumor group. The mean values of age, BMI and smoking time for the LG-cancer group were 58.9±18.51 years (range, 26–81 years), 25.5±3.51 kg/m^2^ (range, 22.2–32.5 kg/m^2^) and 20.6±15.8 packs/year (range, 0–40 packs/year), respectively. These same parameters were calculated for the HG-cancer group as 61.5±12.03 years (range, 43–81 years), 28.1±4.73 kg/m^2^ (range, 20.5–37.8 kg/m^2^) and 25.5±16.94 packs/year (range, 0–60 packs/year) (Table I). There were no statistically significant differences between the patients with LG-UC or HG-cancer with regard to age, BMI and smoking time (P=0.971, P=0.106 and P=0.561).

According to the tumor-node-metastasis classification, there were three (8.8%) patients in the Ta stage, 17 (50%) in T1, five (14.7%) in T2a, one (2.9%) in T3a, four (11.8%) in T3b, three (8.8%) in T4a and one (2.9%) in Tis stage.

### Cytogenetic findings

**Figure 1:**
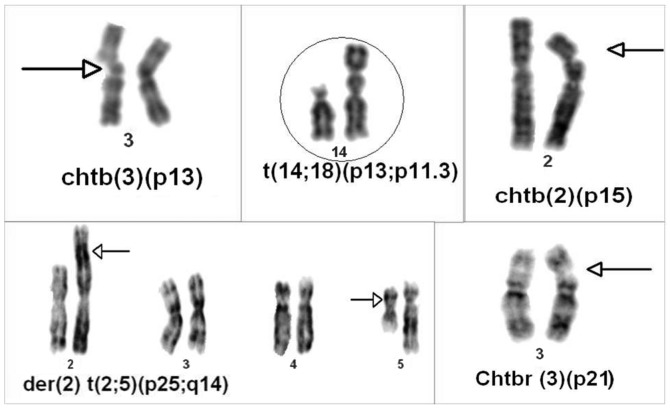
Partial metaphase figures showing chromosomal abnormalities.

CAs were identified in 576 (24.6%) of the 2,344 cells analyzed in peripheral blood [363 (15.5%) and 213 (9.1%) of the cells had structural and numerical aberrations, respectively], and 62 (19.5%) of the 318 cells analyzed in tumoral tissues [24 (7.5%) and 38 (11.9%) of the cells had structural and numerical aberrations, respectively]. Structural aberrations predominated and usually consisted of deletions, translocations, breaks and fragilities in various chromosomes. In particular, deletions in 1p24-pter, 1p32-pter, 1q41-qter, 1q32-qter, 2p13-pter, 2p23-pter, 2p24-pter, 3p13-p14, 3p23-pter, 3p25-pter, 3q11-qter, 5p14-pter, 5q13-q15, 5q31-qter, 7q11-q12, 9q12-qter, 9q22-qterx2, 10q23-qter, 10q24-qter, 11q?, 13q32-qter, 14q11.2-pter, 17q11-qter, 17q21-qter and Xp21-pterx2; translocations between t(2;5)(p25;q14), t(3;12)(p26;q15), t(7;15)(p22;q26), t(7;14)(p15;q24), t(10;14)(q26;q13), t(12;17)(p13;q12), t(13;22)(p11;p11), t(14;22)(q32;q11.2), t(19;19)(q13.4;q13.4) and t(21;22)(p13;p13) ×2; and inversions in izo(Xq/p) and inv(13)(p13;q14) were more frequently observed. In patient 30, inversion of chromosome 9 [inv(9)(p11;q13)] was found in the blood (Table I, Fig. 1). Autosomal monosomies were observed as common findings (chromosomes X, Y, 22, 21, 17 and 8; and trisomies 21, Y, 4 and 15). In the control group, chromosomal aberrations were only found in 33 (2.6%) of 1,250 analyzed cells. The mean number of chromosomal abnormalities in patients with BC compared with the healthy control group was 20±36.2 (range, 0–182) and 1.3±1.6 (range, 0–5), respectively, and the difference between these values was significant (P=0.0001). Also, chromosomal abnormalities were overviewed and compared between the two groups (HG and LG) and almost all of the structural abnormalities found at 1q21, 1q32, 3p21 and 5q31 were detected in patients with HG tumors. Other structural abnormalities were found not only in patients with HG tumors but also in patients with LG tumors.

### FISH findings

**Figure 2:**
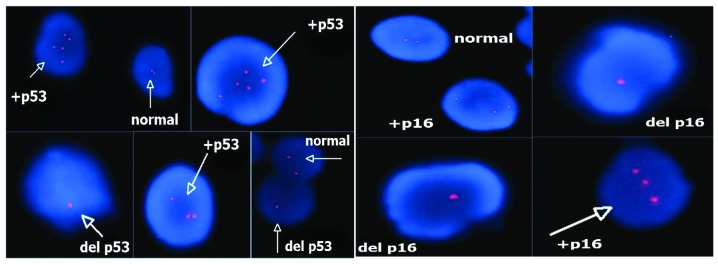
Partial fluorescence *in situ* hybridization images showing amplification of p53 and p16. del, deletion.

A total of 32 patients with multiple copies of the p53 and p16 signals were identified by an interphase FISH screening program using the Poseidon probe. A genetic alteration (amplification and mostly deletion) of p16 was observed in 6.30±4.47 cells (range, 0–60 cells) in the LG group and in 13.8±5.65 cells (range, 0–23 cells) in the HG group, and the difference was significant (P=0.002). Similarly, an alteration (amplification and mostly deletion) of p53 was detected in 7.7±6.21 cells (range, 0–23 cells) in the LG group and 12.4±5.99 cells (range, 0–25 cells) in the HG group, and these differences were also significant (P=0.039). When the cut-off value of 10 altered cells was considered, 19 patients had a positive result for p16 and 17 of these 19 patients had a HG tumor [odds ratio, 13.6; 95% confidence interval (CI), 2.2–85.8]. In addition, 19 patients had a positive result with the same cut-off value for p53, and 16 of these 19 patients had a HG tumor (odds ratio, 6.22; 95% CI, 1.2–32.2) (Table I, Fig. 2).

Although the number of chromosomal abnormalities was higher in the HG group compared with the LG group [23.26±43.19 (range, 0–182) vs. 12.5±4.89 (range, 4–18)], this difference was not significant (P=0.714). However, when the changes of the p16 and p53 genes specifically are considered, these differences were significant (P=0.002 and P=0.039) (Table I).

When all patients were considered, the majority of structural abnormalities were observed on chromosomes 1, 2, 3, 5 and 9, and the majority of numerical abnormalities were observed on chromosomes 8, 17, 21, 22, X and Y. The regions of 1p24–36, 1q21, 1q32, 2q31, 3p21, 3p25–26, 4q31, 5q31, 5q33, 6p21 and 9p-q were detected as being the most affected areas (Table I).

## Discussion

In the present study, the risk factors of age, BMI and smoking time were compared between LGBC and HGBC patient groups. There were no statistically significant differences between the two groups in terms of these factors. Associations between HGBC and older age and longer smoking time were predicted, but no significant differences were found. This may be due to the small study population.

BC is a multistep and complex genetic process and mainly presents as one of two distinct tumor entities: Genetically stable LG tumors and genetically unstable HG tumors (15). While LG tumors are less aggressive, HG tumors can be highly aggressive (6). In the present study, the mean number of chromosomal abnormalities in patients with BC was significantly higher compared with the control group (P=0.0001). In addition, chromosomal abnormalities were detected more frequently in HG tumors compared with LG tumors, but the difference was not significant (P=0.714). Chromosomal abnormalities are more frequently detected in higher-stage than lower-stage BC (16). This means that genetic changes are necessary for the development of cancer and that there is a linear correlation between the aggressiveness of the tumor and the genetic aberrations present.

The 1p24, 1p36, 1q21 and 1q32 regions on chromosome 1 were identified as being the most affected areas in all patients with BC. However, the 1q21 and 1q32 regions were found to be affected more prominently in patients with HGBC compared with LGBC (Table II). A study by Tommasi *et al* (17) isolated the NORE1 gene at 1q32.1 that is homologous to the tumor suppressor gene RASSF1A, and advocated that this gene may be involved with the signal transmission of Ras or Ras-like proteins. Caramazza *et al* (18) reported that specific genes located at 1q21 were associated with myeloproliferative neoplasms, and that this region may contain oncogenes or tumor suppressor genes. According to the results of the present study, the 1q21 and 1q32 regions may contain certain oncogenes or tumor suppressor genes that play a significant role in the development of invasive BC.

Specific alterations were found at 3p21, 3p25 and 3p26 in the patients of the present study. When these findings were compared between patients with HG and LG tumors, the 3p21 loci was dominantly altered in the HG group (Table II). There are a number of reported genes at 3q21 that are associated with either genitourinary or other tumors in the literature. The GPX1 gene was reported as a selenium-dependent detoxifying enzyme gene located at chromosome 3p21, and a study by Ichimura *et al* (19) showed that the GPX1 Pro/Leu genotype was associated with an increased risk of BC and may also be associated with the development of high-stage tumors. The TU3A gene, located on 3p21.2, was reported as a candidate tumor suppressor gene in renal cell carcinoma (RCC). Additionally, Awakura *et al* (20) advocated that this gene is involved in primary cancers of the bladder and testis. The histone methyltransferase gene SETD2/HYPB, located at 3p21.31, was identified as a novel tumor suppressor gene in RCC (21). The RASSF1 gene, located at 3p21.3, is silenced in a variety of human cancers, including lung, bladder, prostate and kidney cancers (22). Jarmalaite *et al* (23) studied promoter hypermethylation of the p16, RARβ, RASSF1A, DAPK and MGMT genes in patients with BC, and hypermethylation of the RASSF1A gene was more frequently detected in muscle-invasive tumors compared with non-invasive tumors. A high frequency of RASSF1A methylation, or the inactivation of RASSF1A, was correlated with an advanced tumor stage and poor prognosis in cases of BC, and hypermethylation of the RASSF1A gene was detected in urine samples with high specificity (24). In conclusion, the 3p21 gene location contains numerous cancer-related genes, and certain genes may be candidates for a panel of markers for BC.

The regions of 5q31 and 5q33 on chromosome 5 were also detected as highly affected areas in the present study, and 5q31 was more frequently altered in patients with a HG tumor rather than LG tumor (Table II). Specific studies have previously reported that in a variety of cancers, certain tumor suppressor genes were located to region 5q31. Dallasso *et al* (25) reported that protocadherin genes that are located to region 5q31 could be tumor suppressor genes in Wilms’ tumor. An association between the sprouty homolog 4 gene at 5q31 and testicular cancer was shown in a study by Kanetsky *et al* (26). These results indicate that the 5q31 gene location requires further study to elucidate its role in BC.

It is known that the p16 gene, located at 9p21, regulates the cell cycle and prevents abnormal cell proliferation. Statistically significant alterations in p16 were detected in HGBC in the present study. The alteration of p16 is concluded to be strongly correlated with the advanced tumor grade. In a previous study, the validity of p16 expression was evaluated in urine cytological and histological samples, and the study reported that a high incidence of p16 overexpression in HGUC was noted in cytological samples and that immunocytological analysis of p16 is a useful method for detecting UC and the tumor infiltrating potential (27). In another study, investigators researched the genetic alterations of the p16 and p14 genes in BC, and they did not find any association between tumor grade/stage and p16 alterations. However, the deletion of the p14 gene was more frequently observed in poorly differentiated tumors. This study also noted that p16 plays a role in early tumorigenesis (28). Conversely, in the present study it was found that the p16 gene was more frequently altered in patients with HGBC. Krüger *et al* (29) assessed the prognostic effect of p16 alterations in patients with T1 stage BC and concluded that there is a significant correlation between the status of p16 and progression-free survival. However, they did not find any significant correlations between p16 status and the tumor grade. The latter finding does not agree with the data of the present study. Currently, there is no consensus regarding p16 status associating to tumor grade, stage and prognosis.

Alterations in the p53 tumor suppressor gene are correlated with a number of varied malignancies. The association between p53 changes and a higher cancer grade, stage, recurrence, progression and mortality has been shown in a number of studies (30,31). Despite these studies, there is conflicting data regarding the p53 status. Malats *et al* (32) overviewed 168 publications from 117 studies and reported that changes in p53 are weakly predictive of recurrence, progression and mortality in BC. In the present study, alterations of p53 were more frequently observed in HGBC rather than LGBC. This difference was statistically significant. Furthermore, this result was similar to that of the p16 gene. Depending on the frequency of p53 alterations in HGBC, the expression of p53 in combination with other markers has also been researched. Shariat *et al* (33) studied four cell cycle regulators (p53, pRb, p21 and p27) in patients with locally advanced BC and advocated that the combination of multiple molecular markers was more informative than examining a single molecular marker. These results indicate that the study of the p53 and p16 genes has had predictive value in the clinic.

Currently used prognostic markers may be inadequate for effective treatment decisions. In the literature, there are numerous studies focused on determining prognostic markers. Although there is currently no consensus about molecular markers for BC, certain genes have been frequently detected in research. In the present study, alterations of p16 and p53 were more frequently detected in HG-cancer patients, and these genes may have predictive values for BC. Aside from these genes, novel chromosomal locations were searched for that may be responsible for the progression of BC. Chromosomal abnormalities of two patient groups were overviewed and compared. Almost all structural abnormalities were detected in the 1q21, 1q32, 3p21 and 5q31 regions in patients with HG tumors. Other structural abnormalities were found not only in patients with HG tumors, but also in patients with LG tumors. Based on this result, it was predicted that these regions may have a significant role in the progression of BC. Aberrations in these areas may be observed as a late event in BC pathogenesis and certain tumor suppressor genes or oncogenes may be located in these regions.

Numerous studies have advocated that the decision of BC management should not be made according to only one prognostic marker. In the present study, the p16 and p53 genes were assessed in patients with BC and it was revealed that these genes were altered more prominently in patients with HG tumors compared with patients with LG tumors, and this difference was statistically significant. In addition to these genes, the structural and numerical abnormalities of chromosomes were also assessed in blood and tissue samples. Certain structural abnormalities were mostly detected in the chromosomal regions of 1q21, 1q32, 3p21 and 5q31 in patients with HG tumors rather than LG tumors. These areas must be further studied to find candidate genes for a panel of BC markers.

## Figures and Tables

**Table I:** Demographic data, p16 and p53 statuses, and blood and tissue culture results of the patients.

P no.	Age^a^/gender	G	S	Tobacco use^b^	p16	p53	RPMI	M199	Tissue
P1	76/M	H	T3b	20	22 del	5 amp 20 del	gap(3p21)	9qh+ ×3;fra(5q31); chbr(14q?);hsr(2q?)x2;45?	47,XY,+2×2; 45,XY,−1;92,XXYY
P2	57/M	L	T1	40	2 amp 4 del	6 amp	92,XXYYx3;fra(1q32);fra(6p21); 47,XY,+21;9qh+ ×2	92,XXYYx4;fra(7q?);45,XY,izo(Xq/p),−21	47,XY,+21;42,XY,−2,−11,−12,−22
P3	61/M	H	T2a	45	10 del	15 del	47,XX,+3p,+ace;45,XX,−14;44, X,−Y, −8; 45,XY,−16; 44,X,−Y,−18,−13,+12	45,XY,−16;45,XY,−8;chtb(1q11); del(10)(q24-qter); 45,X–Y;chrb(2q31); del(17)(q11-qter);45,XY,−22	43,Y,−11,−17,−X;45,XY,−19;45,X,−Y; 44,X,t(14;18)(p11;p11); 45,XY,−13
P4	53/F	H	T1	0	16 del	18 del	9qh+ ×54 48,XX,+4,+21;45,XX−10; 46,XX,?15p+; inv(13)(p13;q14)	9qh+ ×2;44,XX,−12,−21;chbr(4q?); fra(Xq26); 15p+x52; 45,X,−X; 9qh+x2; 15p+,fra(3p25)x2;15p +,chtbr(3p21)x2; fra(12p13); fra(3p21)	46,XX,+17,−20,9qh+;gap(6q15); gap(5q31) ×2; del(3)(p23-pter)
P5	77/M	L	T1	0	3 del	3 del	no cultered	no cultered	no cultered
P6	81/M	L	T1	2	10 del	13 del	9qh+ ×2;fra(4q33);43,X,−1,−10,−Y;45, XY,−17; 44,XY,−21,−22;43,Y,9qh+, −22[2],−X	92,XXYYx2;9qh+ ×2;45,X,−Yx2;45,XY,−20; 44,XY,−3,−18;del(5)(p14-pter),del(X) (p21-pter); chtbr(2q23);chtbr(3p21.3), chtbr(5p13)	no cultered
P7	42/M	L	T1	25	5 del	5 del	45,X-Y ×2;45,XY, −10; del(9)(q12-qter)	del(7)(q11–q12);del(3)(p25-pter);del(5)(q13–q15); 45,XY,−22;chtb(3q26.2);chtb(12q13)	45,X,−Y
P8	73/M	H	T3b	30	16 del	10 del 3 amp	fra(1p36);del(1)(q41-qter);45,XY,−17; hsr(3q11–q13); 45,XY,−3	far(Xp22.1); t(14;22)(q32;q11.2);45,XY, del(17) (q21-qter),−19	47,XYY; 47,XX,+ace
P9	73/M	H	T1	60	9 del	12 del	92,XXYY	45,XY,−22×2;45,XY,gap(1q21)x2,−18;del(13) (q32-qter);9qh+	45,XY,−17;44,XY,−12,−20; 45,X,−Y;chtb(2p15)
P10	53/M	H	T4a	40	18 del	10 del	45,XY,−21; 44,XY,−5,−17	45,XY, chtb(5q15), −18	92, XXYY
P11	38/M	L	T1	10	9 del	12 del	46,XYY,−22; 45,XY,−D 47XY, (?); 45,XY,−14	46,XY, fra(1p36.1); 45,XY, −22; 47,XXY; chbr(9q32); del(3)(p13–p14)	chtb(3q26.2);chtb(12q13)
P12	79/M	L	Ta	0	2 del	5 del	46,XY,−10, +3	46,XY,fra(3p25); 45,XY,−3	45,XX,−21
P13	74/F	H	T1	32	8 del	5 del	45,XX,−12	45,XX,−11×2;48,XX,+15,+16[2],−12;45,XX,−8; 45,XX,−4,−10;43,XX,+15,−8,−10,−19,−21	92,XXXX;92,XXYY;47,XY,+14
P14	50/M	CIS	Tis	30	23 del	18 del	44,XY,−17,−20; 45,XY,−22	45,XY,−8;47,XY,+mar;chtb(3p21.2); chtb(11q13.4)	no cultered
P15	26/M	L	Ta	20	15 del	22 del	45,XY,−22; 45,XY,−8;45,XY,−10; chtb(5q33)	45X,−Y;47,XY,+mar;44,XY,−21,−22;45,XY,−19	45,X,−Yx6;45,X,+4q+,−Y; 43,X,−6, −8,−Y;43,X,−18,−20,−Y; 92,XXYY
P16	81/M	H	T1	0	8 del	10 del	45,XY,−22 ×2; 47,XY, +mar	45,XY,gap(6q23),−14;fra(5q31);fra(2q35); 45,XY, gap(4q27),−Y; 47,XY,+4;45,XY;fra(Xq13), −22; fra(5q31); fra(12q22); fra(Xq26); 45,XY,−15; 45,XY,−22;43,X,−Y,−7,−18; fra(5q31)	del(X)(q21-qter);fra(12q24); del(9) (q22-qter)
P17	51/M	L	T1	20	9 del	6 del	Yq+; chrb(4q31)	47,XY,+21;del(14)(q11.2-pter);fra(11q23);45,XY,−19	no cultered
P18	56/M	L	T1	35	4 del	3 del	44,XY,−18,−21;43,XY,−15,−18,−19;45, XY,−21; chrb(8q22);inv(9)(p11;q12)x3; del(2)(p23-pter)	del(5)(q31-qter);45,X,−8,−9,−Y,+21;47,XY,?+(p); 45,XY,del(1)(p32-pter),−19;del(1)(p24-pter); chrb(8q23); 45,XY,−19	no cultered
P19	44/M	H	T3b	0	9 del 3 amp	6 del 8 amp	t(2;5)(p25;q14); gap(2q23); gap(2q35); gap(4q31.3), gap(5q31); gap(1q36.1); gap(5q31)	45,XY,t(13;22)(p11;p11);45,XY,del(9)(q22-qter),−21; del(2)(p24-pter);t(12;17)(p13;q12);45,XY,−8; 45,XX, −21×2	no cultered
P20	60/F	H	T2a	0	3 del	2 del	47,XX,+mar;del(1)(q32-qter);chtb (2q32.2); 45,XX,−22; chtb(4q31)	t(3;12)(p26;q15);del(10)(q23-qter); 47,XXX; del(3)(q11-qter); 47,XX,+ace	15p+x10;47,XXX,15p+;mar(10<); chtb(12q14)
P21	55/M	H	T1	25	16 del	10 del	45,XY,−22;44,XY,−13,−22;44,XY,−7, −22;45,XY,−13; 45,XY,−16	t(10;14)(q26;q13);47,XY,+15;del(2) (p13-pter);44, XY,+21,−13	92,XXYYx13;92,XXYY,t(19;19) (q13.4;q13.4), t(21;22)(p13;p13); 45, XY,−14; 92,XXYY, t(21;22)XY, −(p13;p13),t(7;15)(p22;q26);45,22; 43,XYY,−2,−4,−8,−15;45,XY,−12; 44,XY,−4,−14
P22	65/M	H	T4	15	12 del	16 del	fra(2q21);chtb(19q13);44,XY,chbr (2q31),−2×2	47,XY,+ace;48,XY,+1,+19,+22;chtb(1q21)(q32)	69,XXYx2;92,XXYYx2;45,XY, −20;chtb(2p15); 45,X,−Y
P23	65/M	L	T1	40	no tissue	no tissue	46,XY	45,XY,−14;45,XY,−8;chtb(5q33)	no cultered
P24	49/M	H	T1	30	no tissue	no tissue	16qh+ ×14	47,XY,+ace;fra(9q32)	no cultered
P25	46/M	H	T2a	30	12 del	7 del	no cultered	46,XY	46,XY
P26	76/M	L	T1	35	0	2 del	46,XY	45,XY,−17×7	no cultered
P27	72/M	H	T4a	45	17 del	14 del	45.X,−Y;chbt (5q33)	45,XY,−8,11p+;47,XY,+21;fra(1q32)x2; fra(1q42); fra(1q22)	no cultered
P28	72/M	H	T3b	20	21 del	18 del	47,XY,+ace;fra(2q33);fra(2q31);fra (3p21);fra(6p21)x2; fra (1q21)x4;22p+x4	45,X,−Y;46,XY,−11,+mar,del(11)(q?); fra(6q23), fra(12q22); fra(2p23)	no cultered
P29	49/M	H	T1	35	14 del	8 del	45,XY,−22; 42,?	45,XY,−9;45,XY,−10;chtb(5q33)	44,XY,−3,−21
P30	79/F	H	T1	0	9 del 3 amp	5 del 4 amp	inv(9)(p11;q13) ×50;45,X ×40; t(7;14)(p15;q24)	inv(9)(p11;q13) ×50;45,X ×40; chtb(16q22)	no cultered
P31	43/M	H	T1	20	0	0	45,XY,−10;43.X,−21,−Y;45,XY,−21; 45.X,−Y	45,XY,−20;45,X,−Yx2;45,XY,−21	no cultered
P32	69/M	H	T2a	30	13 del	12 del	46,XY	46,XY	no cultered
P33	63/M	H	T2a	45	16 del	21 del	chtb(17p11); hsr (2q22) ×3	no cultered	no cultered
P34	54/M	H	T3a	35	12 del	15 del	46,XY	no cultered	no cultered

a package/year.

P, patient; M, male; F, female; G, grade; L, low grade; H, high grade; S, stage; del, deletion; amp, amplification; fra, fragility; chbr, chromosome break; chtb, chromatin break; hsr, homogen staining region; t, translocation; inv, inversion; mar, marker; ace, acentric; ter, terminal; izo, izochromosome.

**Table II:** Comparison of structural and numerical abnormalities for each chromosome in low and high-grade bladder cancers.

Chrom. no.	Structural abnormalities		Numerical abnormalities			
	
	Location (ratio)		Low grade		High grade	
		
	Low grade	High grade	n	Ratio	n	Ratio
1	1p24(1/50), 1p32(1/50), 1p36.1(1/50), 1q32 (1/27)	1p36(1/50), 1q11(1/50), 1q21(7/150), 1q22(1/100), 1q32(4/174), 1q36.1(1/50), 1q41(1/50), 1q42(1/100)	1	(1/32)	2	(2/69)
2	2p23 (1/50), 2q23(1/50)	2p13(1/50), 2p15(2/80), 2p23(1/50), 2p24(1/50), 2p25(1/50), 2q21(1/50), 2q22(3/100), 2q23(1/50), 2q31(3/150), 2q32.2(1/24), 2q33(1/50), 2q35(2/105), 2q?(2/50)	1	(1/12)	4	(4/105)
3	3p13(1/50), 3p21.3(1/50), 3p25(2/100), 3q26.2(2/60)	3p21(6/261), 3p23(1/50), 3p25(2/52), 3p26(1/30), 3q11(1/50),3q11(1/30), +3p(1/50)	3	(3/103)	2	(2/60)
4	4q31(1/6), 4q33(1/32), +4q(1/14)	4q27(1/55), 4q31.3(2/74), 4q(1/52)	0	-	5	(5/234)
5	5p13(1/50), 5p14(1/50), 5q13(1/50), 5q31(1/50), 5q33(2/63)	5q15(1/10), 5q31(8/365), 5q33(2/35)	0	-	1	(1/10)
6	6p21(1/27)	6p21(2/50), 6q15(1/50), 6q23(2/105)	1	(1/14)	0	(0/0)
7	7q11(1/50), 7q?(1/32)	7p15(1/50), 7p22(1/36)	0	-	2	(2/105)
8	8q23(1/50), 8q22(1/50)		4	(4/127)	8	(8/449)
9	9p11(3/50), 9q12(1/50), 9q32(1/50), 9qh+(6/109)	9p11(100/100), 9q22(2/60), 9q32(1/60), 9qh+(64/358)	1	(1/50)	1	(1/10)
10		10q23(1/30), 10q24(1/50), 10q26(1/50)	4	(4/138)	4	(4/170)
11	11q23(1/22)	11q?(1/50), 11q13.4(1/57)	1	(1/12)	4	(4/123)
12	12q13(2/60)	12p13(2/102), 12q14.1(1/50), 12q22(2/105), 12q24(1/10)	1	(1/12)	6	(6/257)
13		13p13(1/54), 13q32(1/52)	0	-	5	(5/220)
14	14q11.2(1/22)	14q32(1/50), 14q?(1/50)	2	(2/40)	5	(5/194)
15		15p+(68/218)	1	(1/50)	5	(5/247)
16		16q22(1/50), 16qh+(14/14)	0	-	4	(4/203)
17		17p11(2/150), 17q21(1/25)	8	(8/39)	6	(6/208)
18			4	(4/164)	4	(4/167)
19		19q13(1/50)	5	(5/205)	4	(4/148)
20			2	(2/64)	4	(4/158)
21			9	(9/268)	10	(10/429)
22		22p+(4/50)	8	(8/292)	15	(15/526)
X	Xp21(1/50), Xq/p(1/32)	Xp22.1(1/50), Xq13(1/55), Xq21(1/10), Xq26(2/107)	2	(2/82)	84	(84/202)
Y	Yq+(1/16)		17	(17/250)	8	(8/382)

Chrom. no., chromosome number; n, number of cells with aberration; -, choromosomal abnormality was not detected.
